# Lectures on Clinical Medicine, Delivered at the Philadelphia Medical Institute

**Published:** 1838-06-06

**Authors:** W. W. Gerhard

**Affiliations:** Physician to the Philadelphia Hospital, &c.


### CLINICAL LECTURES.

LECTURES ON CLINICAL MEDICINE, *delivered at the Philadelphia Medical Institute, by* W. W. Gerhard, M. D., *Physician to the Philadelphia Hospital,* &c.

### ACUTE MENINGITIS.

*Tuesday*, May 15th.—It is my intention, to-day, gentlemen, to continue the discussion of inflammations of the serous membranes, with particular reference to the subject of meningitis. Previously, however, to entering upon this latter topic, I shall call your attention to a case of serous inflammation, which terminated, a day or two ago, at the Hospital, and at the post mortem examination of which, most of you were present yesterday morning. We had, you recollect, acute peritonitis, pervading the whole of the abdomen, the result of a chronic disease of the liver. This disease of the liver was suspected, during the lifetime of the patient, there being sufficient evidence of the enlargement and hardening of the organ. The nature of the affection we found to be cancerous; rounded deposits having the character of vascular sarcoma, were scattered throughout the substance of the liver, offering very fair specimens of this variety of soft cancer. I shall not now enter into an examination of the subject of cancer, but shall confine myself to the acute inflammation of the serous membrane, which was induced by the carcinomatous disease. This case of secondary peritonitis exemplifies the law I enunciated to you, at a previous lecture. Serous inflammations are not dangerous, unless they occur as secondary to a primary lesion of an organ, or are connected with a cachectic state. This secondary inflammation, which is very frequent in the peritoneum, may be either acute or chronic. In the present instance, it was acute, and, probably, arose from the cancerous tumours approaching the surface of the liver. Examples of the chronic secondary inflammation of the peritoneum are most frequent in phthisis, when they are connected with a tuberculous deposit in the serous membrane itself, as was demonstrated to you in one of my last lectures on pathological anatomy.

In the present instance, the peritonitis was acute; it was only within the last two or three days of the patient's life, that he was seized with acute pain over the whole of the abdomen, accompanied with great tenderness on pressure. A tumour was distinctly felt, which I described to you as similar to the pointing of an abscess, and induced me to suspect the existence of suppuration. After the occurrence of the acute pain, the patient sunk rapidly, without any other of the usual symptoms of peritonitis, as vomiting, &c., but his prostration was extremely great. Prostration of this character is a striking symptom of these secondary serous inflammations, and is a most valuable sign in leading us to our diagnosis. You have seen it before in a case of pericarditis succeeding gangrene of the lungs, and in a black man, affected with tuberculous pleurisy. Whenever you have sudden and extreme prostration, supervening upon a chronic disease, in any of the great cavities, you may suspect the existence of secondary inflammation of their serous coats. This was the character of the tubercular peritonitis, depending on perforation of the intestine, noticed in the lecture of the first of May, and I have pointed out to you instances in which there was the same kind of perforation into the cavity of the pleura, following ulceration of the lungs. Perforation is by no means necessary to the production of these secondary inflammations of serous membranes; in the case now before us, the exciting cause was the irritation of the cancerous masses in the liver, but just beneath the peritoneum. The same disease may occur in the ovaries, uterus, and other parts, producing similar results.

Another example of secondary peritonitis, is the affection, generally designated as puerperal fever, a term which is now usually limited to peritonitis, although some physicians are still in the habit of including all febrile diseases of lying-in women under this head. It is imperative, however, to distinguish between these affections. The true secondary peritonitis of puerperal women depends upon the inflammation of the uterus or its veins, or else upon the irritation consequent upon delivery, but it is rendered more frequent and more severe by a strong tendency to suppuration, which extends to all the membranes and organs of puerperal women, and gives rise to the various affections which are sometimes called puerperal fever.

The anatomical signs, in this case, were similar to those observed in the other cases of serous inflammation, which have come under your notice just now, from their great prevalence in spring and the beginning of summer. Thus, to study pathology, you see how necessary it is to pass through cycles of disease. In these serous inflammations, you see how interlocked they are with all other diseases, occurring sometimes as idiopathic, but, in the large majority of cases, as secondary, being rarely fatal, in the first instance, except when attacking the membranes of the brain. Continuing the subject of special serous inflammations, I shall now proceed to take up the subject of *meningitis*.

Meningitis may be easily confounded with other affections of the brain. We had a case a few weeks ago, of a surgical patient, affected with disease of the urethra, in which it was with some difficulty that we made out, even after death, a satisfactory diagnosis, the point being settled with certainty, merely by the presence of a slight quantity of pus. I was called to the case, only a short time before the man's death, when the only striking symptom was delirium, which I looked upon as merely the concluding act of life. The true nature of the affection was, however, revealed by a post mortem examination. We found, first, a bright injection of the pia mater, which is characteristic of inflammation, particularly, if there be no serum present. Injection of the large vessels is not indicative of inflammation, but merely of conjestion, the two not usually co-existing together, a bright arterial tint denoting the one, while the other gives a dark blue color to the surface implicated. The injection was, in this case, spread over the whole surface of the membrane ; this is generally the case, although it predominates at one portion, either the base or the summit of the brain. Here, the inflammation predominated at the summit, involving the faculties of the intelligence, while, in children, it usually occupies the base, and is connected with a disturbance of the senses. The distinction I make here, coincides with one of the leading points of phrenology, which allots the faculties of the intellect and of the senses to different portions of the brain. Although I look upon the details of this science, as still founded only upon the imagination, yet the great fact, that the intellect is connected with the summit, and the senses with the base of the brain, is unquestionably true, and confirmed by pathological observations.

The roughness of the serous coat, the arachnoid membrane, is the next point to be noticed in this case. It might seem, that mere effusion of liquid would be enough to characterize inflammation of this membrane. This is not so, however; when it is in a healthy state, there exists a liquid, which is clear and transparent, which in the early stages of real inflammation, becomes altered in quality and deficient in quantity. The inflammation is not so much of the arachnoid membrane, as of the subjacent pia mater, in the meshes of which the morbid products are chiefly retained. True inflammation of the arachnoid is of very rare occurrence. In the present case, we found pus, mixed with lymph in the pia mater, giving a yellowish appearance to the membrane. The three great features, then, of this case, from which we concluded that it was one of acute meningitis, were the injection of the small vessels of the arachnoid, the roughening of this membrane, and the deposit of lymph and pus beneath it.

The consideration of this case, offers another point of much interest—the connection between affections of the urinary organs and diseases of the brain. Ten years since, my attention was first directed to this subject, upon observing a man, labouring under stricture and thickening of the lining membrane of the urethra, to my great astonishment, perish suddenly from cerebral symptoms. At the Pennsylvania Hospital, two or three years ago, I noticed the death of a man from similar symptoms of disease of the brain, after a few days illness, who had been previously suffering from inflammation of the neck of the bladder and urethra. Various writers, and particularly Lallemand, have called attention to this subject. Dr. Lallemand has dwelt, specially, upon the connection between diurnal seminal emissions dependent upon chronic inflammation, and the development of cerebral disease. The cases of this character, described by Lallemand, he usually traced to chronic gonorrhœa, which, occasioning a thickening of the neck of the bladder, the vesiculæ seminales, and the ductus ejaculatorius, left the latter in a patulous condition, allowing a discharge of semen to take place without ejaculation, during the acts of urining or fecating. The dependence of cerebral disease upon causes of this nature, is a highly important fact, and which will assist you in understanding some affections, otherwise not easily explanable. The seminal weakness, of long continuance, enfeebles the understanding, and, finally, the brain is disordered to such an extent, that medical relief is sought for. I have had several cases of this character, in which the affection was supposed to be connected with a nervous temperament, was, in short, referred to various other causes than the correct one, but, in every instance, I was able to make out the previous existence of chronic gonorrhœa, producing the condition of the urino-genital organs, which I have described, and, through this means, giving use to functional cerebro-nervous disturbance. M. Lallemand attempts to cure these affections by directing his treatment to the urinary organs.

In the case of the man at the Pennsylvania Hospital, to whose death, with cerebral symptoms, I have alluded, we found upon examination after death, the vesiculæ seminales and ductus ejaculatorius destroyed, an abscess behind the verumontanum, filled with pus, and the coats of the bladder contracted and thickened. The particular history of the case was not taken, but it illustrates finely, how chronic diseases of the urethra give rise to affections of the brain, and how causes, trivial in themselves, may produce serious and fatal functional disturbances. Death, to use the words of M. Lallemand, may be the result of a series of illness, dating their origin from an attack of gonorrhœa; the importance of curing without delay this affection here, of course, impresses itself upon you.

From this digression, I return to the subject of inflammation of the membranes of the brain. The case of the man Brown, which has been under your notice for some time past, at the hospital, will serve as a fair illustration of the subject. We have no example in the hospital of acute meningitis, but the case of Brown, which is of the sub-acute form, being more slow in its progress, and better marked in its character, will very properly serve as introductory to the study of the acute type of the disease. This man was taken ill with cephalalgia, in the region of the forehead and frontal sinuses. We inferred, as we had a right to, that it was not a case of secondary meningitis, from the absence of any previous ill health. Soon after the commencement of the headach, the senses became implicated; the sight of the left eye was impaired, and the hearing was disturbed with tinnitus, buzzing, resembling the noise produced by a saw, and, as the affection declined, it was like the humming of bees. These comparisons are the patient's own expressions, and were not elicited by any leading question; they are, therefore, the more descriptive of the symptoms. With the advancement of the disease, there were dulness, sadness, and somnolency, but no delirium. There was contraction about the eyebrows and the root of the nose, forming, as I mentioned when noticing this symptom in the lecture on tubercular meningitis, one of the best marked signs of meningeal inflammation. This contraction was, in this case, of a permanent character, and would have enabled any one, at all accustomed to the affection, at once to recognise it. There was no paralysis; no subsultus. The inflammation was confined to the anterior and inferior parts of the brain, not extending to the summit, as the faculties of the intelligence were but little impaired, nor was there much lesion of the cerebral substance, for their was neither paralysis nor rigidity.

After establishing the symptoms, the question starts itself, with what affections might this case be confounded? With very few. First, it could not be acute meningitis. The tongue was natural, and, although, there was some constipation, there was no nausea or vomiting; there was no cough; nor was there any unnatural excitement of the pulse. The inflammation was then limited to a small spot of the brain, for, had it been more extended, the pulse must have shown it, by becoming unduly excited. The same absence of paralysis which showed that there could be but little cerebral lesion, would indicate that the disease did not depend upon large tubercles, or other chronic tumours of the membranes, for these lesions speedily produce palsy. By way of exclusion, therefore, we succeeded in localizing the affection, and we recognised meningitis attacking the anterior portion and base of the brain. In addition to its anti-febrile character, its course, which lasted a month, a much longer duration than belongs to the acute form of the affection, and its gradual decline, satisfactorily demonstrated its sub-acute progress.

The prognosis was an important point, which came up for discussion, at the period of the man's entrance into the hospital. It was at first doubtful; was the meningitis secondary, and dependent on the presence of a tumour of tubercles, or the like? After the lapse of two or three days, it was clear that there was no chronic disease, but that the affection was a mere local phrenitis or meningitis. We made our minds up to this conclusion, from the evident absence of all symptoms of an impaired constitution. The man had not been ill before the time of his recent attack, he had never called for the aid of a physician, nor had his friends; I say his friends, because in chronic cerebral affections the patient himself is often afraid to call attention to his symptoms, and the first application for medical relief is on the part of his friends, as the evidence of some decided mental aberration is forced upon them.

The treatment proper in acute meningitis, is sufficiently well exemplified by that pursued in this case. There are certain great laws well laid down for the management of this affection, which are much more clearly understood, than the subject of therapeutics in general, owing, I think, to the fact, that close observation is more easy in meningitis from its rapid and well defined symptoms, than in diseases of the thorax or abdomen. The following are the points to be attended to in treating acute meningitis.

Blood-letting, in patients who give evidence of tolerable strength, embonpoint, and previous good health, is always advisable, particularly so in the acute form of the disease, when depletion becomes a measure of absolute necessity, and if it be neglected your chance of saving your patient is but small. Should your bleeding be large or small? It is best, I think, to take a considerable quantity of blood at once from the patient, if he be a stout and healthy man; you may thus, sometimes, immediately arrest the disease. When serving in the Pennsylvania Hospital, I had a case illustrative of the good effects of this practice, and of the great importance of a correct diagnosis in cerebral affections. A man was brought into the cells, said to be labouring under mania a potu: he was a sailor who had just made a voyage from Boston; he had been drinking to excess, but had also been working hard exposed to a very hot sun. Upon examination, I found the signs he exhibited to be not those of ordinary mania a potu; his head was hot, his pulse quick, in short, he was in the first stage of acute inflammation of the brain. I bled him largely, between twenty and thirty ounces, and he was, I may say, instantly cured. I, indeed, the next day directed a slight cupping, a purge and the like, but they were merely by way of precaution. Had this patient been treated by opiates as a case of mania a potu, he must almost infallibly have died. Such cases I have seen treated in this manner in hospitals, for so common a vice is drunkenness in this country, that all diseases of the brain occurring in intemperate persons, are apt to be indiscriminately treated as the effects of excessive indulgence in ardent spirits. When I was a resident physician in the Alms House Hospital, a woman was brought in with a fracture of the skull, upon which arachnitis supervened. She was treated as a case of mania a potu, by a gentleman who was writing a thesis on this subject, whose mind was consequently absorbed by this single variety of cerebral affection. She died, and upon examination after death, spicula of bone were found driven in upon the dura mater!

After you have bled once largely, it is best to limit a repetition of general bleeding to cases of individuals of a very plethoric habit. In place of general depletion, keep up cupping and leeching, which, if persisted in with pertinacity, will do much good. In cupping, every thing depends upon the manner in which it is applied. In the case of Brown, all the cuppings were of service except one, when the cups were applied to the temples; here it seemed only to augment the irritation—the pressure of the cups very near the seat of disease causing an afflux of blood to the part. The cuppings,which were addressed to the back of the neck, all did good. I do not speak of cups to the forehead, because no body thinks of using them in that quarter. My advice, then is to cup rarely to the temples, and generally to the back of the neck; leeches behind the ears may be employed with much advantage; in this very case, I found leeching behind the ears of service, when the cupping ceased to do good, showing the mere change in the manner of abstracting blood to be of essential importance. Leeching, then, is to be sometimes resorted to, though cups are generally to be preferred in taking blood locally, from the ease with which the quantity may be regulated, and the facility with which they may be obtained.

In very acute meningitis, you have within your control another powerful remedy, and one that is quite as important as any of the others—ice to the head. It is to be applied with caution and you are to judge of its producing its effect by the supervention of faintness, languor, or paleness of the face. In hospitals, the ice may be applied in a tranquillizing chair, but in private practice, where you have no such convenience, a bandage may be employed for this purpose; you must be careful to remove the application as soon as the ice melts, otherwise the alternation of heat and cold thus produced, may do harm. The use of ice I would continue for several days, until there was a decided abatement of the acute symptoms. It is a great point in the management of this remedy, to have for the patient proper attendance of persons who can control him. For this purpose one, two, or three men nurses will be indispensable in private practice, where those means of restraint are wanting, which are to be met with in lunatic hospitals.

The next remedy I shall mention, acts on the same principle as the last, and is intended to produce revulsion from the head; it is the application of warmth and stimulating poultices to the extremities. I was treating a patient some time ago with ice to the head, in whom, although, the ice was evidently doing good, as it produced pallor and languor, the symptoms, abated but little; upon examination, finding the feet cold, I directed warm stockings to be put on them, had sinapisms applied, and ordered them to be occasionally plunged into warm water, which was followed by an evident amelioration of the symptoms. Unless you attend to these precautions, you will lose much of the good that may be derived from the application of cold to the head. Upon trifles like this, success in a great measure depends, in the management of this affection; indeed in therapeutics the advantage which one practitioner has over another, depends chiefly upon his attention to minute and seemingly unimportant details.

Although it may be somewhat irrelevant, I cannot here forget to caution you against falling into those habits of careless and hasty prescribing, which are sometimes produced by a negligent attention to the practice of public institutions. The advantages of hospitals are inestimable to one who uses them in a right spirit; that is, as schools of diagnosis, and of the great therapeutic indications. But you must remember that in private practice you must carefully direct or even superintend in person, a multitude of details, which are usually attended to in hospitals by well-trained nurses, aided by the system which exists in all well conducted institutions.

Much of the reputation of Dr. Physick as a practical physician, depended on a strict attention to these minute points of detail, and he had, therefore, often better success in the management of even medical cases, than persons who were perhaps more familiar with pathology, but not equally attentive to these particulars.

Purging is a remedy which has been almost from time immemorial adopted in the treatment of acute inflammations of the brain. The saline purgatives combined with senna, or a mercurial purge, are those generally employed. I prefer a mercurial purge, as it serves a double object, by acting on the liver, and preparing the way for ptyalism, if it should afterwards become necessary; it is besides a good preparation for the saline articles. I would begin by ten grains of calomel, followed up the next day by a dose of salts and senna; should the calomel not purge, it will salivate, which is not to be dreaded. After, then, a single mercurial purge, you may give doses of senna and salts,—a robust patient will require half an ounce of each; these, by inducing serous discharges from the bowels, will have a derivative effect. Afterwards your object should not be to produce violent purging, but to keep up a moderate looseness of the bowels.

Should the delirium not yield to depletion and purging, these remedies should not be continued after the strength of the patient begins to decline, but you must now have recourse to mercury, in small doses, and to blisters. Mercurials, like tartarized antimony, act as antagonists to inflammation, and may with propriety be employed in the second stage, or in the sub-acute form of the affection. They would have been highly appropriate in the case of Brown, in whom we should have prescribed them, had the disease not yielded in the first instance to the local depletory treatment. It is best to continue the use of mercury until ptyalism is produced. By effecting this, I have succeeded in curing a large proportion of the cases which have occurred in my wards of the hospital. An interesting case happened last summer, which, perhaps, some of you may recollect. It was that of a young man, who had been a clerk at Mobile, and who on his way to Philadelphia, by the Mississippi river, had been taken ill with fever and delirium at Cincinnati, from which he recovered with difficulty. He came to Philadelphia, not quite well, having still some symptoms of cerebral disease. He was taken ill again, and brought to the hospital. He was then in a state of high cerebral excitement, being occasionally rational and relapsing again into delirium; throughout the night he would be in a state of great liveliness, loquacious, restless, with his senses considerably exalted. From the history of the case, I concluded it to be one originally of acute meningitis, which had now become chronic, and began the treatment of it with blisters and local depletion, but the delirium did not yield, until the effects of a mild mercurial course were produced. Another case I may mention was that of a young sailor, who was taken ill under circumstances which I do not now recollect. He had pleurisy first, and afterwards meningitis, and the disease did not abate till after a mercurial course. The symptoms were not the violent delirium of the last mentioned patient, but mere stupor, dulness of the senses, and constant disposition to throw his head strongly backwards. Neither of these cases was dependent on the presence of tubercles or other chronic lesion.

We come next to speak of blisters, which, it might seem at first sight, would be proper at an early period of the affection. This is not the case, however; in the first stage, they seem only to irritate, and decidedly augment the extreme agitation and violent delirium; they should be delayed till the acute symptoms subside, when they may be applied over the occipital region, extending to the back of the neck. They are to be rarely applied over the whole scalp, where they give great pain. The same law that regulates the employment of leeching or cupping is applicable here; the blisters do more good, at some distance from, than immediately over the inflamed portion of the brain; when the disease is more chronic, it is often useful to keep a blister discharging behind each ear, as I have already advised in the treatment of acute hydrocephalus.

Caustic issues or incisions over the fontanelles have been recommended in chronic meningitis, but as they would be very inconvenient, they have not been generally used, though I see no reason why they should not be employed in certain cases, especially where there is reason to apprehend that the disease has followed an injury of the head.

The plan of treatment which I have gone over, will succeed in curing the majority of cases of acute meningitis. If, however, the affection should not yield, and passes into the chronic state, the patient remains necessarily more or less insane, and is apt to sink into the third stage of insanity, or dementia. He becomes utterly incoherent, and the case usually terminates in a very curious but totally incurable variety of paralysis, called the paralysis of the insane.

In the beginning of this kind of insanity, when the appearances of active inflammation have in a great degree subsided, cold affusions upon the head, repeated several times daily, mild laxatives, a sparing diet, abstinence from all excitement or exposure to the sun, with gentle exercise, prove the most useful remedies. In short, the treatment must be extremely mild, but persevere while a hope remains of saving your patient from the worst species of insanity.

When acute meningitis is fatal, the patient generally dies at the end of the second, or in the third stage of the disease, or he may die from meningeal apoplexy. I have twice or thrice seen a patient in the Alms House, labouring under meningitis, become suddenly comatose, with stertorous breathing and loss of power of the limbs. The symptoms were those of apoplexy, arising from effusion of blood, not into the substance of the brain, but, on the surface of both hemispheres into the membranes, and which from its pressure is, therefore, necessarily fatal.

Whether the inflammation of the membranes of the brain be acute or not, as the third stage, or that of effusion of lymph or pus supervenes, the delirium becomes less violent, the disturbance of the senses is succeeded by a total abolition of them, the patient neither seeing nor feeling. There is a gradual supervention of paralysis; sudden dilatation of the pupils in place of alternate contractions; and there is usually, but not always, strabismus. This stage is necessarily fatal, there being no possibility of a recovery.

To recapitulate briefly the course of my remarks to-day—you have had your attention called to the anatomical characters of certain serous inflammations, and after tracing the connection between cerebral affections and those of the genito-urinary organs, we have entered at length into the treatment of acute meningitis, basing my remarks upon a case of the sub-acute variety which has been lately under notice at the hospital. I have not gone into details of the symptoms of acute meningitis, waiting till they present themselves to our notice, which from our knowledge of the law attending the appearance of these affections, must be the case during the course of the summer. The sub-acute variety is the only one which I am now able to demonstrate, although my therapeutic observations chiefly relate to more acute forms.

---

### CHRONIC MENINGITIS—APOPLEXY, &c.

*Tuesday, May* 23d. At my last lecture, I continued the subject of inflammations of the brain, and entered particularly into that of acute meningitis, which I was able to illustrate by a case of the sub-acute form of the affection, at that time under your notice at the hospital. I merely alluded at the time to the subject of chronic meningitis, without entering into it at any length, and I, therefore, propose now, to say a few words upon it, as it properly belongs to this period of my course. We have a large number of cases of this affection in the wards of the hospital. I shall select the best marked of them, that of Urweiler, a German, to whose history and symptoms I shall briefly call your attention. This man, two or three years ago, having previously enjoyed good health, received a blow on the head, the effects of which, at the time, were not very seriously felt. He suffered slight headach, pain, &c., which, however, soon abated. But, after a lapse of time, the powers of his mind began to fail, and he became, finally, entirely deranged, and in addition to this disorder of the intellect, paralysis is gradually supervening. This latter symptom, as you may have observed in the hospital, is a very common accompaniment of insanity, chiefly of dementia; it is, however, often met with in persons in whom the insanity is not yet developed, the functions of motility being attacked before the intellect is much impaired. The disease is, therefore, often to be recognised at first by the mere disorder in the powers of movement, and may ordinarily be detected as follows. Slight symptoms of insanity are presented, often not well marked, but, again, rapidly becoming strongly characterized, and running into the worst degree of madness, incoherence. The organs of locomotion become also affected, the first symptoms being a failure in the power of walking, and feebleness of the upper extremities rarely at first occurring; a hobble or limp is noticed, generally, at first, on one side of the body only. Other changes then take place, the upper extremities becoming involved, the face slightly distorted, the tongue is protruded with difficulty, and the speech thick; these symptoms, however, are often indistinct, with the exception of the failure in the power of walking, which always shows itself. The symptoms, for the most part, gradually but slowly advance, scarcely ever retrograding. If the patient is insane when this partial paralysis appears, the affection is nearly always fatal. Dr. Calmeil, who was connected with the lunatic hospital, at Charenton, near Paris, considered it always fatal; and my own prognosis is always more or less of the same character. It is somewhat singular that this disease is much more frequent in men than women; although very common at the Bicêtre and Charenton, it is quite rare at the Salpetrière where none but women are admitted.

I shall not now enter at length into the pathological features of the affection, merely bringing before you two cases, that came under my notice some time ago. One was of a gentleman, who died about two years since; he had been hurt by a fall, from the consequences of which he seemed to have recovered; but, two or three years subsequently, his walking began to fail, soon afterwards his mind, and a short time only passed after the development of these symptoms, before he died. On examination after death, we found the membranes of the brain universally thickened. The other case was that of a man, who had been a master of a vessel in the merchant service; just previous to his attack, he had been suffering from a soreness of throat, which improved but little under a treatment consisting chiefly in local applications; symptoms of disease of the brain soon appeared, and the man entered the Pennsylvania Hospital. At this time, he had incomplete paralysis of the lower extremities, and of the left arm, with painful deglutition; these symptoms went on slowly, but, finally, destroying the patient. After death, we detected a slight thickening of the membranes lining the ventricles, and were astonished to find how little the medullary substance of the brain was affected, and that the cortical substance was merely in the normal state.

Treatment in chronic meningitis is available only when the functions of motility are not impaired, and those of the intellect alone are affected. The mode of treatment to be resorted to, consists in a regulated diet, blisters to the nape of the neck and behind the ears, and cold affusions twice or thrice a day. Although I cannot affirm that I have cured any patients labouring under actual paralysis, I have, certainly, by the plan detailed to you, restored several in whom the disease had proceeded no further than the affection of the mind. The patient Urweiler is much better, speaks with greater ease, and has obviously more strength in the limbs.

The next disease of the brain, which I shall notice, offers, at this time, several cases in the wards of the hospital, and will be often encountered by you, in the course of your practice—I mean *apoplexy*. Of the cases in the hospital, one is a recent one, and two, in the black wards, occurred as far back as a year ago. The term, apoplexy, is often used very indefinitely. I shall here employ it to signify an actual hæmorrhage into the substance or beneath the membranes of the brain, excluding mere effusions of serum, all cases where there is no organic lesion of the brain, as well as those in which mere congestion occurs, an affection which is most frequent during the summer season, and at the close of the winter. These cases are all confounded with true apoplexy, and indiscriminately classed under the same name; sometimes, indeed, when no one function of the brain is disordered, the term apoplexy is given to sudden deaths. A man will fall down dead, perhaps, with some comatose symptoms from disease of the heart, and his death is at once referred to apoplexy; whereas, genuine apoplexy almost never causes instantaneous death. When the case terminates fatally, it is usually after a lapse of some months, from paralysis. It sometimes proves fatal in the course of a few minutes, or half an hour; but in these cases there is usually blood effused into the ventricles, and it is not common for it to terminate before the end of several hours, even when most severe. The exceptions to the rule, that sudden death does not follow apoplexy, are indeed so rare, that you may pretty safely pronounce an instantaneous death to be independent of this cause. These very sudden deaths are usually owing to diseases of the heart, although, in some of them no organic lesion whatever can be found of any organ.* There was an example of this a few months since at the hospital, in a patient who was labouring under a chronic disease of the heart, who died during my visit. I found him in his ordinary condition, and had just left the ward, when I was suddenly called back and found him dead.

The anatomical characters of apoplexy are easily ascertained, and may be divided into two great varieties—in the first and most common, the effusion of blood takes place into the substance of the brain, in the other it takes place into the membranes. Any spot in the brain may be the seat of the hæmorrhage, but it is generally the thalamus of the optic nerves and corpus striatum. The blood is sometimes poured out in such quantity as to break into the ventricles, and even force asunder the septum between them, so that it presses upon both hemispheres of the brain; but it is generally confined to a single spot, on one hemisphere.

The character of the clot is always the same; it consists of a mass of dark coagulated blood, surrounded by the tissue of the brain, which is, to a certain extent, ecchymosed and softened: this softening may be either the effects of previous disease, or the consequence of the apoplexy. Dr. Rochoux, who observed at the Bicêtre hospital, thinks that

* Memoir of Dr. Louis.

apoplexy always depends upon the previous existence of local softening in the brain, the hæmorrhage afterwards taking place in the diseased portion. My own, and the general opinion is, that in the large majority of cases, softening of the brain around the clot is a consequence of the pressure from the blood thrown out, the hæmorrhage itself, depending upon a disorder of the circulation. The cause of the deranged circulation is sometimes hypertrophy of the heart, which increases the impetus of the blood; at other times, the cause is to be sought for in a diseased state of the arteries and capillary vessels. But although this opinion of Dr. Rochoux is too exclusive, it is by no means unfounded, for there is a certain if not a large proportion of cases, in which the evidence is decidedly in favor of previous lesion of the cerebral substance. These cases are somewhat analogous to the hæmorrhage which follows diseased uterus, or the advanced stages of pulmonary tubercles.

After the clot has been some time in contact with the substance of the brain, it is in a measure isolated by the formation of a cyst which completely surrounds it. It is afterwards gradually absorbed, absorption taking place in the following order: first, the serum disappears; secondly, the colouring matter; and, thirdly, the fibrine of the blood. After a lapse of some months, the cyst only remains, in one of two conditions:—it is either entirely hollow, and lined with a new serous coat, or a little cellular substance occupies the old seat of the apoplexy. In one of these two forms, the parts are invariably found.

This succession of lesions has been perfectly well illustrated, by the cases which have been just now under our notice, for there are particular symptoms, corresponding to each stage of the disorder. One was that of the old woman, in ward No. IV., in whom there was a complication also of softening of the brain. For, in addition to the paralysis which follows hæmorrhage, we have strong contraction of the flexor muscles on the paralyzed side, so violent, that pain is given to the patient by an attempt to extend them. She had been well, we learned, two weeks before the attack, which determines it at once to have been of an acute character. The next point would be the manner in which it occurred,—was it sudden or gradual? This we cannot settle satisfactorily, from our inability to ascertain the previous history of the case.

The paralysis might arise either from acute softening or apoplexy, and as the distinctive characters of these two affections chiefly depend upon the abrupt commencement of the latter, and the more gradual progress of the former, it is evident that we cannot make a positive diagnosis. The rigidity which is so striking in the paralyzed side of the body, is produced by softening, but this softening may be merely secondary to the hæmorrhage. It does not take place in the beginning of apoplexy, until the parts around the clot become inflamed, whereas, in inflammation of the brain, the numbness, stiffness, and rigidity follow in rapid succession, from the onset of the affection, and precede perfect paralysis. There is another reason for regarding the case as apoplexy; that is, the extent of the paralysis, which is rarely so great in inflammation. In acute and sub-acute meningitis, as I have before remarked, attacking the summit of the brain, you have delirium of a more or less violent character, the mind being always compromised; if it involves the base of the brain, you have alteration of the functions of motion, as subsultus, spasms, &c., and the sight is affected, but, as there is no paralysis at first, you cannot confound these diseases with apoplexy.

Another point of some importance, in making a diagnosis of apoplexy, is to distinguish it, during the first few hours of the affection, from mere congestion of the brain. This is not so easy at first, as after the lapse of a few hours; but there are some peculiarities about these affections, which, if closely attended to, will serve to draw the line of distinction. In the first place, after the first few minutes of the loss of consciousness, which usually occurs at the beginning of both, there is paralysis of one side alone, in apoplexy. In congestion, on the other hand, there is scarcely ever complete paralysis of either, but there is generally some difficulty of motion in both. Persons attacked with apoplexy, are not so commonly of the same full habit of body, as those who suffer from active congestion, so that this plethora is alone sufficient to induce you to suspect the case congestion. A nice diagnosis, at the beginning of the two affections, is not very important; it is only after they have advanced beyond the first stage, that it becomes of consequence, as regards the treatment, to distinguish between the two. For every case, offering the symptoms of loss of consciousness, difficult breathing, turgescence of the vessels of the face, &c., but one course of treatment is to be thought of, the actively depletory. But after the subsidence of these immediate symptoms, cases which thus far offered the same character, demand a widely different plan of treatment.

I have alluded to cases of effusion of serum on the brain, which are sometimes confounded with apoplexy from hæmorrhage, and are termed serous apoplexy—a term often used to denote the presence of comatose symptoms, without hæmorrhage. This serous apoplexy may occur from the effusion of serum beneath the membranes, or into the ventricles of the brain; in the latter case, it is not infrequent in mania a potu, and also in some diseases of a chronic character. These cases, however, of comatose symptoms from serous effusion are of rare occurrence, except at the close of cerebral diseases of easy diagnosis.

Sudden coma, entirely independent of organic disease of the brain, sometimes appears, as the consequence of a previous chronic disease of various viscera, or even of mere anemia, under circumstances calculated not a little to puzzle the practitioner. A case of this sort occurred the winter before last, in my wards of the hospital. A seaman, who had been exposed to great hardships, and had contracted a disease of the liver in the East Indies, of which he bore well-marked evidence in a pale, yellow, jaundiced skin, came into the ward complaining of neuralgic pains in the feet, unattended with fever. He had no symptoms whatever of disorder of the brain, or of the thorax; nor of the abdomen, except those indicative of a diseased liver. After remaining for a short time in the hospital, he was one night found with comatose symptoms, stertorous breathing, &c., having been seen, only an hour before, walking acrcss the ward for a cup of water. I saw him only an hour before his death, in a state in which it was exceedingly difficult to arrive at a correct diagnosis; I, however, came to the conclusion that it was not apoplexy, from the fact of the symptoms not being limited to one side of the body. An examination after death revealed no alteration whatever of the brain, except a very trivial quantity of serum beneath the arachnoid. He had, therefore, coma, loss of consciousness, and stertorous breathing, during life, without any lesion of the brain.

Symptoms of the same character occur from the effects of heat upon the nervous system, during the warm season. During the intensely hot weather of the summer of 1830, I witnessed the opening of the bodies of twenty or thirty persons, who died from this cause; we found no organic disease of the brain, but merely a slight congestion, such as is observed in other acute diseases, which it would be idle to set down as a cause of death. These were the appearances only in those who died suddenly of exposure to heat; for if time elapses for reaction to come on, inflammation of the brain may take place, but only as a secondary affection.

The other two cases of apoplexy, occurring in the hospital, to which I shall direct your notice, offer varieties of the disease, different from the first described. They were black men who came into the wards, in a state of complete paralysis of one side of the body, one of them scarcely able to speak. He could articulate but the monosyllable, *no*, which he answered to all questions whatever that were put to him. He seemed conscious of the ridiculous nature of this invariable answer, but could not increase his vocabulary for several months, when he was gradually able to pronounce the shorter words, and now speaks very well, although there is still paralysis of one side of the body. In the other, the speech was merely thick, but his mind remained tolerably clear. They continued in this state for several weeks, and as the process of the absorption of the clot advanced, the intelligence brightened, but the paralysis remained. Our cases were hemiplegia, one side of the body being affected; that, opposite to the side of the brain, in which the hæmorrhage occurred. This latter conclusion we make from a law of pathology to that effect, which is almost without an exception, in its operation. There may be one, two, or three abnormal cases out of a thousand, but, in making your conclusion, you may safely leave them out of the estimate.

As the next consideration, in our diagnosis, we had not only paralysis of the lower portion of the body, but also of the upper extremity, and the muscles of the face, with disturbance of the intellect and senses, establishing, of course, the seat of disease to be the brain. The stiffness of the limbs was gradual in its progress, caused by inflammation around the clot; but the paralysis was, at the time of the patient's entrance, perfect; and the mouth was drawn towards the side which was *not* paralyzed, which is the reverse of what occurs in cerebral inflammation where the paralysis is active, that is, the mouth is drawn towards the palsied side. Our diagnosis and prognosis were at once made out; there was a hæmorrhage on one side of the brain, and it was incurable, because the paralysis was complete, in which cases it is for life, the only chance of recovery being when it is incomplete.

The liability of apoplexy to return is a matter of notoriety, and a point perfectly well understood in the world; you should, therefore, in all cases where it has once occurred, be on the watch, looking for a recurrence of the hæmorrhage, which nearly always takes place near the same spot, between tbe thalamus and corpus striatum.

The cases under notice were not fair specimens for testing the treatment proper for apoplexy; but some of you may recollect a case, which occurred last summer, of incomplete paralysis in a woman, which yielded entirely to treatment in a week. Dr. Forille of Rouen, explains the different success of the treatment in cases of paralysis from hæmorrhage by the occurrence in some cases of an actual rupture of the fibres of the brain, while in others these fibres are merely separated by the effused blood without being actually torn. I am myself inclined to this opinion, and believe that the medullary fibres are actually broken in most cases of complete hemiplegia. The routine of treatment in apoplexy is simple and familiar to all medical men. Very free bleeding is of course indispensable, in all patients, who are at all plethoric ; if of a very pale complexion, it is to be practised with some care. Purging, foot-baths, and cupping are to be resorted to, although the latter is not of the same value here as in meningitis, where it is our sheet-anchor. I here indicate merely the general outline of treatment, to be pursued in apoplexy, not entering into any details on the subject. In regard to depletion, I may remark that it is a point of some delicacy to determine how far to carry it. My rule is, to continue depleting until the circulation in the vessels of the head is lessened, which is to be ascertained as well from the appearance of the eye and countenance as from the pulse. Purging I also push to some extent; but you must be careful not to purge too violently, or that state of chronic softening of the mucous membrane of the intestinal canal, which was mentioned in a previous lecture as a frequent accompaniment of the exanthemata, may occur; it is a most unpleasant complication in paralytics, who rarely resist a diarrhœa long, however much they may have been previously benefited by purges. Blisters, setons, and issues have all been used in apoplexy, but with indifferent success, although the keeping up of a discharge, by these means, is excessively useful in chronic meningitis. If, however, the apoplectic symptoms are pertinacious, these remedies may be tried once or twice, and continued according to the effect produced.

If you are called to a patient, suffering from apoplexy, after the full mischief of the hæmorrhage is produced, and *perfect* paralysis is established, it is your duty to announce at once to the friends of the patient the impossibility of his ultimate recovery, explaining to them the nature and amount of organic lesions existing in the brain, and the impossibility of an entire cure.

The last point in the treatment of apoplexy to which your attention must be directed, are the sores which are likely to occur about the sacrum, trochanters, &c., if the patient is obliged to keep his bed for any length of time. The bladder is also apt to become diseased, in this affection, and you must watch and guard against too long a retention of the urine.

I shall conclude this lecture, by saying a very few words on the subject of acute softening of the brain. This affection is to be known from apoplexy, by the presence of fever, dizziness, and vertigo, while you will rarely observe any febrile movement, in the cerebral hæmorrhage, till some time after the effusion of blood has taken place. The numbness of the limbs, which is present in softening of the brain, comes on very gradually, and, although the intellect is feeble from the first, yet the impairment of its faculties is comparatively slow in its advance, there being at first, and for some time, merely dulness, and no active delirium afterwards. In a black man, under my care, four years ago, at the Pennsylvania Hospital, the delirium assumed the character of well-marked mania. This maniacal delirium is different from the more active kind occurring during the inflammation of the membranes and cortical substance of the brain. It is an affection which rarely occurs, except in the young and middle-aged, and is not to be classed with chronic softening of the brain, which is a sort of necrosis, or gangrene of this organ, and met with only in old persons. In this latter disease, there is no active febrile movement whatever, the patient advancing, with unfailing certainty, from bad to worse, to death. The affection is dependent, according to Dr. Carswell, on a cartilaginous condition of the blood vessels. Dr. Rostan, of the Salpêtrière Hospital, observed the disease on a large scale, and has published a monograph upon the subject, in which he states it to be beyond the reach of treatment. In this country, I regret to say, that our experience does not materially differ from that of Dr. Rostan.

I here conclude my remarks on the organic diseases of the brain. There still remain numerous functional disorders of this organ, to which we may perhaps allude on a future occasion.

